# Commentary: Donepezil enhances understanding of degraded speech in Alzheimer's disease

**DOI:** 10.3389/fnagi.2018.00197

**Published:** 2018-07-13

**Authors:** Tom A. Campbell, John E. Marsh

**Affiliations:** ^1^Department of Psychology and Logopedics, Faculty of Medicine, University of Helsinki, Helsinki, Finland; ^2^Department of Building, Energy and Environmental Engineering, University of Gävle, Gävle, Sweden; ^3^School of Psychology, University of Central Lancashire, Preston, United Kingdom

**Keywords:** cognitive hearing science, Donepezil (aricept), sinewave speech perception, Alzheimer's dementia (AD), new early filter model

Speech communication under adverse conditions (Mattys et al., 2012; Marsh et al., 2015) requires the interaction of hearing with cognition, upon which the new cognitive hearing science centers (Arlinger et al., 2009). Adverse conditions affecting speech communication include cognitive impairments (Mattys et al., 2012), which could relate to age- and disease-related cholinergic decline (Zubieta et al., 2001; Terry and Buccafusco, 2003). Such declines concern the role of the ligand acetylcholine in the transfer of information within the brain. Cholinergic decline is more of a consequence than a cause of Alzheimer's dementia. The cause is neurodegeneration via complex mechanisms with underpinnings in multiple genomic, proteomic, and metabolomic cascades (Cacabelos, 2007). However, in palliative treatment, clinical doses of drugs known as acetylcholinesterase inhibitors, including Donepezil (Cacabelos, 2007), can delay the progression of the cognitive symptoms of Alzheimer's dementia by a matter of months (Francis et al., 1999). These drugs can improve the ease and efficiency by which cognitive working memory operates (Furey et al., 1997). Working memory arguably supports the storage of contextual information necessary for perceptual decision-making during the concurrent processing of speech information that is obscured or degraded.

Hardy et al.'s recent approach to degrading such information is to use sinewave speech: Sinusoids, which follow the center frequencies of the original utterance's formants, trace only modes of the supralaryngeal vocal tract resonant frequency (Remez et al., 1981). Such sinewave replicas exclude all other speech information. Prior to Hardy et al.'s investigation, it remained unknown whether enhanced acetylcholine transmission would improve the top-down perception of degraded speech. In persons with Alzheimer's dementia, using Donepezil, Hardy et al. showed exactly that.

Vesicles of the neurotransmitter acetylcholine convey information from neuron-to-neuron (Figure 1A). Reductions in the number of such vesicles can impair that informational transfer (Figure 1B). Acetylcholinesterase is an enzyme catalytic to the neurotransmitter's hydrolysis (Figure 1C). Acetylcholinesterase inhibitors, such as Donepezil, are drugs that reduce the rate of this breakdown by biochemically interfering with acetylcholinesterase's action (Figure 1D). The consequence is slow hydrolysis (Figure 1E). A nervous system with acetylcholine shortage exhibits suboptimal synaptic signaling between neurons (Figure 1B). Acetylcholinesterase inhibitors thus offer systemic control of acetylcholine levels (Figure 1D).

**Figure 1:**
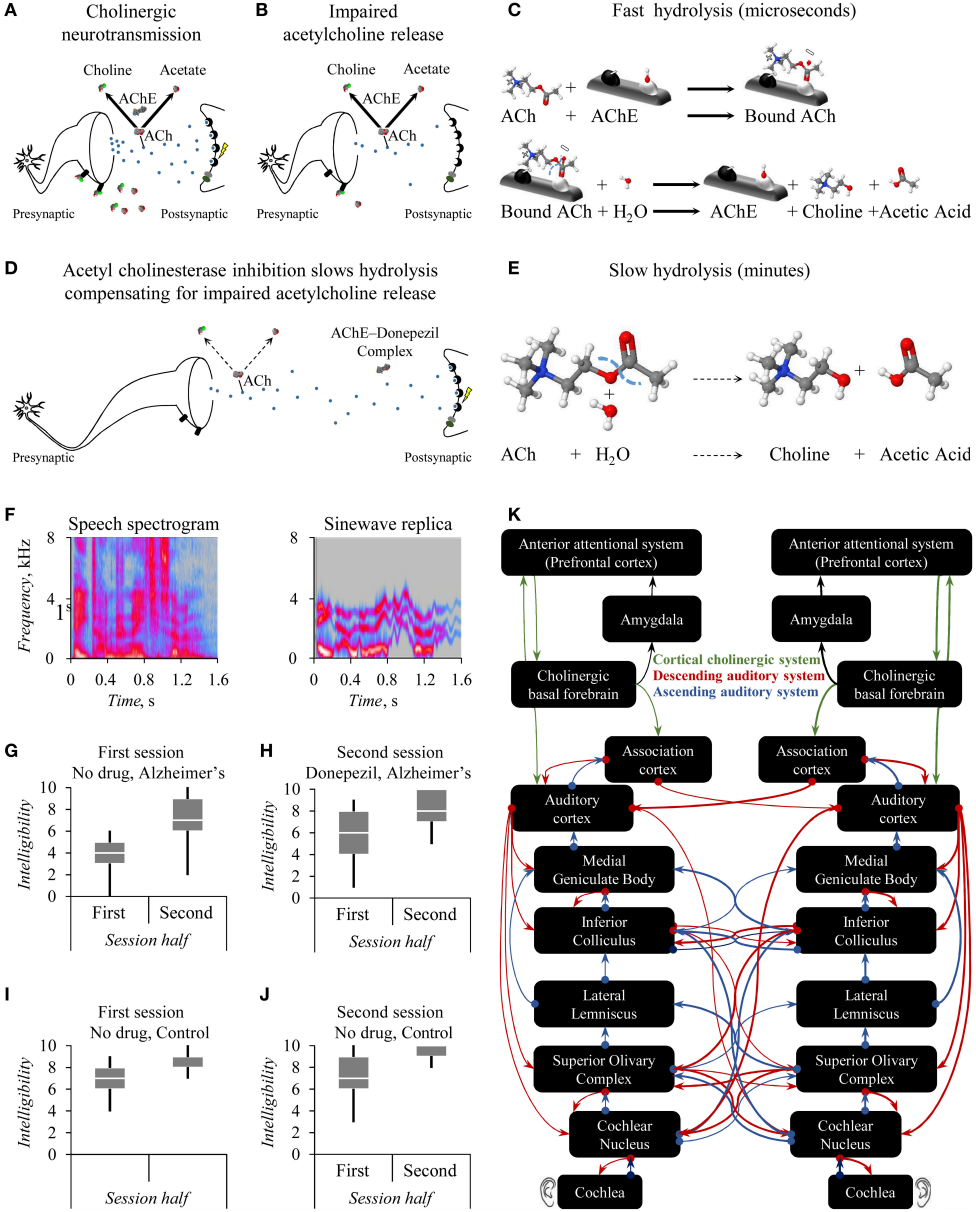
Theory of how acetylcholinesterase improves intelligibility of sinewave speech in persons with Alzheimer's dementia. Cholinergic neurotransmission **(A)** can exhibit age- or disease-related abnormalities with consequences for neurotransmission including impaired acetylcholine release **(B)**. This impairment yields less acetate for presynaptic high-affinity choline transporters to supply intracellular synthesis of the acetylcholine ligand (ACh) for packaging into vesicles to release into the synaptic cleft **(B)**. In turn, there are fewer such vesicles of acetylcholine to bind to postsynaptic muscarinic receptors **(B)**. Binding to muscarinic receptors indirectly increases intracellular Ca^+^ levels initiating a postsynaptic action potential **(A)** that does not occur with fewer vesicles and thus less such binding **(B)**. A distinct mechanism to initiate action potentials relies on the binding of ACh to nicotinic acetylcholine receptors permitting the influx of extracellular Ca^+^. This neurotransmitter ACh undergoes a fast hydrolysis that the macromolecule acetylcholinesterase (AChE) rapidly catalyzes into choline and acetic acid **(C)** that yields acetate ions in extracellular equilibrium **(A,B)**. The acetylcholinesterase inhibitor Donepezil forms an AChE-Donepezil complex **(D)** blocking gorges in AChE macromolecules containing the illustrated active anionic and esteric sites to which ACh would otherwise bind during fast hydrolysis **(C)**. This acetylcholinesterase inhibition in turn can upregulate impaired cholinergic neurotransmission **(D)** by slowing hydrolysis **(E)**. The perception of sinewave speech **(F)** improves during a session **(G,H)** from baseline levels **(G)** following a clinical dose of Donepezil **(H)** for persons with Alzheimer's dementia. The extent of this drug effect exceeds any null influence of practice across successive sessions for their age-matched controls **(I,J)**. The interpretation offered **(K)** is that the prefrontal cortex of the cortical cholinergic attentional system improves the otherwise impaired control of the auditory cortex via the cholinergic basal forebrain. Theoretically, the basal forebrain top-down controls an early filter, which affects speech perception within loops of the brain's interacting ascending and descending auditory systems including the rostral brainstem **(F–H)**. *Credits*: Copyright 2017 by CDC Global (https://flic.kr/ps/2mHk9t2mHk9t2mHk9t2mHk9t). Reprinted courtesy of Hardy et al. under a Creative Commons License CC BY 4.0 (https://creativecommons.org/licenses/by/4.0/).

Hardy et al. presented persons with Alzheimer's disease and healthy elderly control adults with either clear speech or sinewave replicas. These stimuli were 3-digit lists such as “eight hundred and eighty-seven” (Figure 1F). To-be-attended speech information was not obscured by to-be-ignored sound, as occurs with speech in noise. Rather, in such sinewave replicas this speech information was instead degraded. At first, most listeners perceive those degraded speech-derived replicas as pitch-varying whistles. However, after some exposure, a top-down insight occurs: The listener starts to hear the message as speech.

Having Alzheimer's disease hampers perception of the degraded sinewave speech (Figure 1G cf. Figure 1I) yet not the clear speech. Both during a first baseline session and during a second listening, whistles become more speech-like: Both groups show improvements in degraded speech perception within each session (Figures 1G–J). However, administering Donepezil between sessions to persons with Alzheimer's disease further improves degraded speech perception (Figures 1G, H). Enhanced acetylcholine neurotransmission thus seems to improve this perception of degraded speech for persons with Alzheimer's disease whom tend to have disease-related cholinergic abnormalities. Comparable effects do not generalize to other cognitive tasks, so Donepezil affects neurocognitive mechanisms closely allied to speech perception.

An extant cognitive hearing science account of the influence of selective auditory attention on speech perception under adverse conditions adopts a cholinergic top-down control assumption (Marsh and Campbell, 2016) with which Hardy et al.'s drug effect harmonizes: The limited set of possible three-digit utterances promotes top-down processing. This processing uses the stored memory of the immediately preceding acoustical context during the concurrent proactive prediction of the utterance. That processing also uses the stored memory of information gathered in favor of lexical candidates to retroactively repair perceptual decisions. The concurrent processing during the storage of these memories is a working memory function. Acetylcholinesterase inhibition can improve processing efficiency: Physostigmine not only reduces the effort required to perform a working memory task but also the regional cerebral bloodflow in the right prefrontal cortex (Furey et al., 1997). With Donepezil, treating cholinergic abnormalities, theoretically, the prefrontal cortex of the cortical cholinergic attentional system (Sarter et al., 2005) better controls the auditory cortex. Such top-down control takes place via the cholinergic basal forebrain enhancing degraded speech perception.

An unexplored corollary of this cholinergic top-down control assumption is that the cholinergic cortical attentional system, including the prefrontal cortices, controls an early filter (Sarter et al., 2005; Marsh and Campbell, 2016). Accordingly, prefrontal control of loops of the brain's interacting ascending and descending auditory systems realize this filter. A top-down modulation affects structures of the rostral brainstem early on within the bottom-up ascension of auditory information (Figure 1K). Mild cognitive impairment causes abnormalities in the auditory responses of the human rostral brainstem, which could reflect initial signs of conversion to Alzheimer's disease (Bidelman et al., 2017). An untested prediction is thus that pharmacological manipulations of top-down cholinergic mechanisms would affect subcortical speech processing.

Hardy et al.'s findings thus unleash the potential of a new cognitive hearing science. This science could establish how acetylcholine affects speech-in-noise performance in persons with hearing or cognitive impairments: Empirically undetermined is whether Hardy et al.'s cholinergic influence confines to a top-down focusing of the listening to attended speech information; be that signal degraded or obscured. During speech-in-noise perception and understanding, another cholinergic influence may also be upon the top-down selective inhibition of ascending to-be-ignored noise (Petersen et al., 2017). Uninhibited, such noise arguably causes distraction by diverting a prefrontal capacity limitation (Marsh and Campbell, 2016) away from the to-be-attended speech information that the noise also masks.

Some sobering words of caution are necessary: Without the design administering Donepezil to healthy controls, a question germane to the cholinergic top-down control assumption goes unaddressed. Could there be a cholinergic influence on speech perception in persons without Alzheimer's Disease? Hardy et al. do provide normative data that tend toward ceiling (Figure 1J), in which performance on a more demanding speech perception task, or pupillometry, may prove even more revealing (Wendt et al., 2017) administering Donepezil to such healthy elderly persons. A design with an additional placebo-controlled arm would also better substantiate Donepezil's influence on speech perception (Hardy et al., 2017), were the drug effect to replicate with a significantly stronger between-sessions improvement than in a placebo-control arm. Further, different acetylcholinesterase inhibitors show different affinities for and selectivity to acetylcholine, yet remain about as therapeutically effective (Cacabelos, 2007). Different acetylcholinesterase inhibitors thus have distinct collateral mechanisms of action that are indirectly related, or even unrelated, to cholinergic neurotransmission. Whether some such collateral mechanisms mediate Hardy et al.'s effect of Donepezil remains unknown: Experimental comparisons with different acetylcholinesterase inhibitors are lacking.

Turning from theoretical caution to conservative assays of clinical potential, a subtle breakdown of the brain's network for language comprehension predicts the conversion of individuals with mild cognitive impairment into persons with Alzheimer's disease (Mazaheri et al., 2018). Regular activity of this language comprehension system may thus be paramount. Germane is that having hearing loss associates with increased risk of dementia (Lin et al., 2011). Hearing aid use eliminates this association (Amieva et al., 2018). Using Donepezil associates with reduced mortality for nursing home residents with dementia (Gasper et al., 2005). Contrastingly, chronic administration of another acetylcholinesterase inhibitor, Galantamine, increases mortality (Kirshner, 2005). Given the known side effects (Kirshner, 2005), for Donepezil to become a viable treatment for cognitive difficulties with speech intelligibility under adverse conditions, there thus remain objectives for responsible clinical research: Without any chronic administration increasing mortality risk, long-term effects on intelligibility should be shown that eliminate longitudinal associations between hearing loss and the subsequent incidence or progress of Alzheimer's disease.

## Author contributions

Both JM and TC made substantial contributions to the concept and interpretation in drafting the manuscript, approved the submitted materials, and have agreed to be accountable for all aspects of the work in ensuring that questions related to the accuracy or integrity of any part of the work are appropriately investigated and resolved.

### Conflict of interest statement

The authors declare that the research was conducted in the absence of any commercial or financial relationships that could be construed as a potential conflict of interest.
